# The Metropolitan Press and the Hospitals

**Published:** 1898-04-09

**Authors:** 


### The Hospital

A Journal of

The Medical Sciences and Hospital Administration.

Vol. XXIV.—No. 602.] Edited by Sir Henry Burdett, K.C.B., and Solomon C. Smith, M.D., M.R.C.P. [April 9, 1898.

### THE METROPOLITAN PRESS AND THE HOSPITALS.

The outside public have very little idea of the burden of responsibility which rests upon the editor of a metropolitan daily paper. No other country can be compared with our own so far as the press is concerned. In Great Britain, although we have an ably conducted, independent, and influential provincial press, the London daily newspaper is, after all, the chief centre of information to which the whole of the people look, and which the more intelligent of them take in, in addition to the local papers. It follows that there is nothing too great or too small to demand the consideration of the London editor. It is not generally understood that the income of the greater charities which have their headquarters in London is upwards of seven millions sterling per annum. Of course, charity, and especially those branches of charity which immediately affect the social wellbeing of the people, must engage the attention of the metropolitan press. Yet the enormous number of institutions to be dealt with constitutes a difficulty so great that an editor cannot treat the subject at length in the ordinary course, owing to the exigencies of space. From this very circumstance, every well-managed institution, and especially the hospitals and medical charities, owes much to the metropolitan editors, who are always willing to the fullest extent of their power to help every good work of this kind. We can never forget the substantial service rendered to the hospitals by the leading morning, evening, and weekly papers in London in 1895, when they succeeded in arousing and fixing public attention on the Hospital Sunday Fund to an extent never before equalled in its history. The results achieved were satisfactory, as such results always must be when those who are able to form or lead public opinion combine to aid any cause which is of vital interest to the wellbeing of the community at large.

What would the social life of the poor in a large city like London be without the hospitals? It is the fashion in certain quarters to attack the casualty departments of the larger hospitals, and to point to them as sinks of iniquity, because so many trivial ailments or accident cases are there dealt with every year. We have spent hours in the casualty departments, and the oftener we visit them the more are we impressed with the feeling that the work which they do is so grateful to the feelings of the poorer citizens, and so exactly meets the necessities of the masses, that their existence under proper regulations is essential to the orderliness and quietude of the metropolis. Hospitals are not now regarded as they were thirty years ago. At that time people were accustomed to look upon a hospital as a place of refuge, to which accidents and disagreeable cases were removed out of sight, and which wise people refrained from entering, especially if they were sick. All this has been changed, thanks in large measure to Lord Lister and his system, to the improved nursing and general hygiene and construction of the hospitals, and the fact cannot be denied that people, when they are seriously injured or sick, feel that after all there is no place for them like a hospital. This feeling is highly creditable to those responsible for the management of the hospitals and to the members of the medical profession, and it has gradually formed public opinion so that hospitals are now recognised as an important part of the social economy which must be adequately maintained in the best interests of everybody. Of course, powerful as the press is, it cannot do everything; but there is much that it can do, and ought to do, for London, which cannot be done at a reasonable cost with thorough efficiency without the co-operation of the daily newspapers.

It gives us very great pleasure to announce that Mrs. Spender, the wife of the editor of the *Westminster Gazette*, who has for years been a most valuable visitor to the wards of the London Hospital, is organising a press bazaar to be held at the Hotel Cecil in June next in aid of the largest hospital in London. She has already secured the co-operation of several metropolitan newspapers, and we have no doubt, from what has already happened, that at this bazaar every metropolitan daily newspaper of importance will have its own stall. We have consented to organise such a stall, and we are confident that the readers of The Hospital will co-operate in making it one of the most interesting of all. Of course, the sympathies of many readers are necessarily directed to one particular institution, but on an occasion like the present some time and energy may surely be spared for the sake of the principle which will underlie this united action of the metropolitan press in recognition of the importance of maintaining our voluntary hospitals upon an adequate basis. By taking part in this movement everybody should feel that they are showing their appreciation of the spirit which the responsible editors of the metropolitan press have invariably displayed by helping every worthy hospital in the time of its special need. Mrs. Spender has great organising powers, untiring energy, and a kindly influence begotten of enthusiasm for a good cause. These gifts should secure to her not only the support of the press, but the co-operation of every man and woman in London who has leisure or means to give. By helping Mrs. Spender everybody can give expression to the gratitude which all owe to the hospitals, and show their appreciation of the importance of this movement, made by our colleagues in the lay press, in support of medical institutions.

